# Phytochemical Constituents and Biological Activities of *Ononis spinosa*: A Comprehensive Review

**DOI:** 10.3390/plants15091409

**Published:** 2026-05-05

**Authors:** Vlad-Ionuț Nechita, Alexia-Paula Tărău, Angie-Ioana Şuster, Mihaela-Ancuța Nechita, Anca Toiu, Daniela Benedec, Daniela Hanganu, Costel Siserman, Cristina Drugan, Ilioara Oniga

**Affiliations:** 1 Department of Fundamental Sciences, Discipline of Medical Informatics, Research Methodology and Data Analysis, Faculty of Nursing and Health Sciences (FAMSS), “Iuliu Hațieganu” University of Medicine and Pharmacy, 400012 Cluj-Napoca, Romania; nechita.vlad@umfcluj.ro; 2 Faculty of Medicine, “Iuliu Hațieganu” University of Medicine and Pharmacy, 400012 Cluj-Napoca, Romania; 3 Department of Pharmacognosy, Faculty of Pharmacy, “Iuliu Hațieganu” University of Medicine and Pharmacy, 400012 Cluj-Napoca, Romania; nechita_mihaela_ancuta@elearn.umfcluj.ro (M.-A.N.); atoiu@umfcluj.ro (A.T.); dbenedec@umfcluj.ro (D.B.); dhanganu@umfcluj.ro (D.H.); ioniga@umfcluj.ro (I.O.); 4 Department of Forensic Medicine, Faculty of Medicine, “Iuliu Hațieganu” University of Medicine and Pharmacy, 400006 Cluj-Napoca, Romania; 5 Department of Medical Biochemistry, Faculty of Medicine, “Iuliu Hațieganu” University of Medicine and Pharmacy, 400012 Cluj-Napoca, Romania

**Keywords:** *Ononis spinosa*, phenylpropanoids, flavonoids, secondary metabolites, biological activity, phytochemistry

## Abstract

*Ononis spinosa* L. (Fabaceae), commonly known as spiny restharrow, is a widely distributed medicinal plant traditionally used in European and Middle Eastern phytotherapy, particularly for the management of urological and inflammatory conditions. Despite its long-standing ethnomedicinal relevance, comprehensive syntheses of its phytochemical profile and biological activities remain limited. This review aimed to summarize current evidence regarding the chemical constituents and pharmacological effects of *O. spinosa*. Four electronic databases (PubMed, Scopus, Web of Science, and SpringerLink) were searched for studies published between 1997 and 2024. The search yielded 308 records; after duplicate removal and eligibility screening, 34 studies met the inclusion criteria. The phytochemical profile of *O. spinosa* is characterized predominantly by isoflavonoids (e.g., ononin and other formononetin derivatives), triterpenes, phenolic acids, and additional polyphenolic compounds. Although the phytochemical profile of *O. spinosa* includes multiple classes of secondary metabolites, this review places particular emphasis on phenolic compounds, given their prevalence and well-documented biological activities. Experimental evidence indicates a broad spectrum of biological activities, including anti-inflammatory effects (associated with cPLA2α inhibition and cytokine modulation), antibacterial and antifungal activity, antioxidant capacity, wound-healing and dermatological benefits, as well as diuretic and anti-adhesive effects in urinary models. Additional reported properties include antiproliferative, anti-adipogenic, analgesic, and neurotrophic activities. Proposed mechanisms of action involve enzyme inhibition (e.g., Hyal-1 and COX-2), modulation of transient receptor potential (TRP) channels, redox regulation, and interference with microbial adhesion and inflammatory signaling pathways. Overall, *O. spinosa* contains bioactive compounds exhibiting a wide range of pharmacological activities supported by in vitro and in vivo studies. Among the investigated effects, anti-inflammatory, urological, and wound-healing activities appear to be the most promising targets for future research. These findings highlight its therapeutic potential while emphasizing the need for well-designed clinical studies to further validate its medicinal applications.

## 1. Introduction

**Figure 1:**
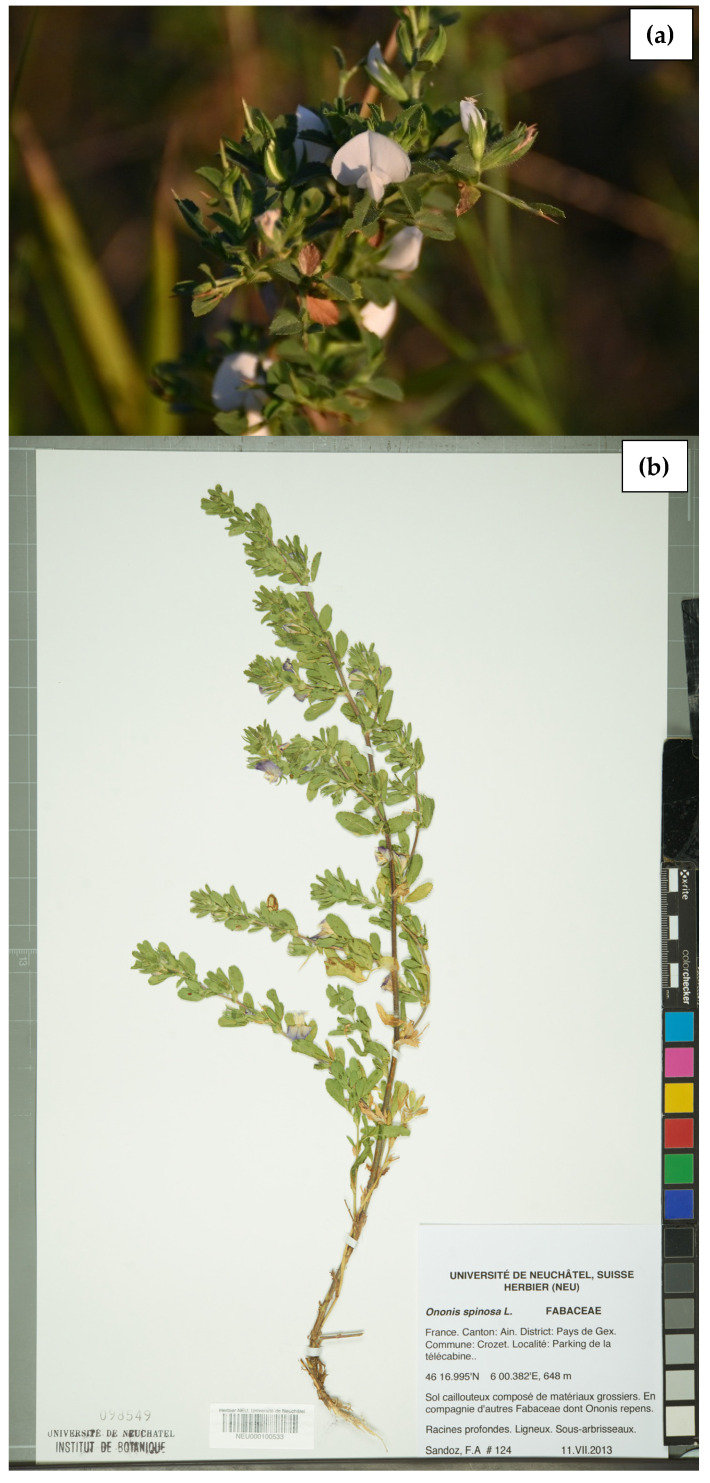
*Ononis spinosa*: (**a**) aerial parts; (**b**) whole plant (herbarium specimen). **Notes:** (**a**) Photograph of *Ononis spinosa* aerial parts photographed in Romania, Hagieni, Constanța County (44.06° N, 27.82° E; July 2024), courtesy of Ruxandra Popa, reproduced with permission. (**b**) Herbarium specimen image obtained from Wikimedia Commons (Neuchâtel Herbarium), licensed under CC BY-SA 3.0.

*Ononis spinosa* (L.) (Figure 1) is a flowering and fruit-bearing plant, part of the family Fabaceae, which also includes widely known food crops such as peas, beans, lentils, and chickpeas. Commonly known as “spiny restharrow”, *O. spinosa* is taxonomically classified within the kingdom Plantae, clade Tracheophytes, clade Angiosperms, order Fabales, family Fabaceae, subfamily Faboideae, genus *Ononis*, and species *O. spinosa* [1,2,3,4].

The Fabaceae (Leguminosae) is the third-largest family of angiosperms, comprising over 750 genera and approximately 19,500 species, following Asteraceae and Orchidaceae [5,6,7,8]. It includes herbaceous plants, shrubs, and tree species, with herbaceous forms particularly diverse in temperate regions and woody species characteristic of tropical ecosystems [6,8,9,10].

As for *O. spinosa*, the species occurs mainly near sea level, but it can also be found at elevations of up to 2000 m [11,12,13]. The plant is widely spread across Europe, North Africa, and parts of Western and Central Asia [7,14].

*O. spinosa* is a short to tall subshrub characterized by two rows of hairs and stiffly upright, spiny stems that do not root below. Most of the leaves are trifoliate, linear to oval, and unnotched. Flowers are 15–20 mm in loose racemes, pink or reddish purple in color [15]. Fruit pods are hairy, oval, and split along the central line.

The lack of rhizomes, more upright habit, darker blooms, and the presence of two lines of hairs up the stem rather than hairs all around can also be used to distinguish it [4]. Across its distribution range, the species is primarily pollinated by bees [11].

Studies have suggested that the bioactive constituents of *O. spinosa* contribute to both systemic and topical therapeutic effects across a range of disorders (Stojković et al., 2020) [16,17,18]. Traditionally, different extracts of *O. spinosa* have been used externally for the management of wounds and inflammatory skin conditions [17,19]. Beneficial effects have also been reported following internal administration, in the treatment of rheumatism, inflammatory conditions, and urinary tract infections [17,20].

Different parts of the plant have been utilized for distinct therapeutic purposes. The flowers, leaves, and roots have been traditionally employed for their lithontriptic and diuretic properties, while extracts obtained from the roots and aerial parts have been used in traditional medicine for their antitussive and mild laxative activities [21].

Further investigations have reported antimicrobial activity of *O. spinosa* extracts against both Gram-negative and Gram-positive bacteria, including *Pseudomonas aeruginosa*, *Escherichia coli*, and *Staphylococcus aureus*, as well as yeast species such as *Candida albicans*, *C. glabrata*, and *C. krusei* [22]. In addition to their antimicrobial potential, the anti-inflammatory properties of *O. spinosa* have also been investigated. For instance, the ethanolic root extract may reduce experimentally induced edema in rat models under specific experimental conditions [23]. One proposed mechanism underlying this anti-inflammatory activity involves the inhibition of cytosolic phospholipase A2α (cPLA2α), as suggested for the methanolic extract of *O. spinosa* [24].

Additional studies have investigated cosmetic anti-aging [25] and analgesic effects [26], while antioxidant and antiproliferative activities have also been reported for *O. spinosa* extracts [16]. Hepatoprotective activity has been investigated; however, no protective effect was demonstrated, and experimental findings even suggested a potential adverse influence on liver function [26].

Phytochemical analyses indicate that *O. spinosa* contains a wide range of secondary metabolites, primarily derived from the phenylpropanoid biosynthetic pathway. The most abundant constituents are flavonoids, especially isoflavones, which occur in both free and glycosylated forms. Identified compounds include ononin (formononetin 7-O-glucoside), genistin (genistein 7-O-β-D-glucoside), formononetin, biochanin A (4′-methylgenistein), and biochanin A 7-O-glucoside 6″-O-malonate. In addition to flavonoids, phenolic acids derived from hydroxybenzoic and hydroxycinnamic acid pathways have also been reported, including p-hydroxybenzoic, vanillic, caffeic, salicylic, and gentisic acids. Other classes of secondary metabolites such as tannins further contribute to the polyphenolic profile of the plant. Beyond phenolic constituents, extracts of *O. spinosa* have also been shown to contain volatile oils, triterpenoid saponins, deoxybenzoin derivatives such as ononetin, lectins, homopipecolic acid, and various mineral components [21,27,28].

The aim of this review is to synthesize the available evidence regarding the phytochemical composition and biological activities of *O. spinosa*. Despite its wide geographical distribution and long-standing use in European traditional medicine, particularly in urological disorders, comprehensive integrative reviews focusing on this species remain limited. Therefore, this work compiles and critically discusses the available literature on its major bioactive constituents, pharmacological properties, and proposed mechanisms of action, providing a consolidated scientific perspective on its medicinal potential.

## 2. Materials and Methods

This study was conducted as a comprehensive narrative review of the literature on *O. spinosa*, covering references published between 1997 and 2024. Literature research was performed across four electronic databases—PubMed, Scopus, Web of Science, and SpringerLink—using a unified keyword-based strategy, adapted to the specific syntax of each database. The core search query was: (“*Ononis spinosa*”) AND (“chemical composition” OR “phytochem*” OR “therapeutic*” OR “pharmacolog*” OR “bioactiv*” OR “metabolite*” OR “extract*”). The primary literature search was conducted up to July 2024. Additional relevant articles published thereafter were included during manuscript preparation. Searches were limited to records published in English and restricted to research Articles. All retrieved records were subsequently screened for relevance based on title, abstract, and full-text evaluation. Study selection was performed by two independent reviewers, and any discrepancies were resolved through discussion.

**Figure 2:**
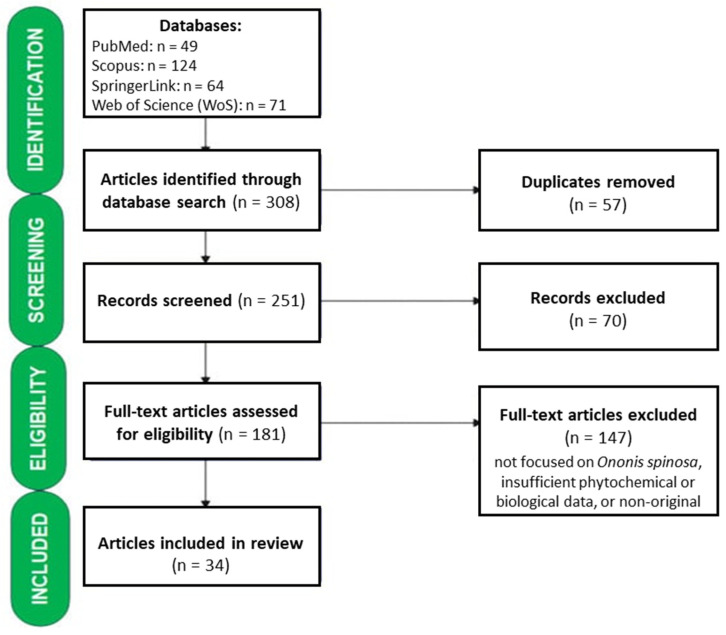
Literature selection workflow and study screening process. **Note:** Studies were excluded at the full-text stage when they did not meet the inclusion criteria, particularly due to lack of relevance to *Ononis spinosa*, insufficient phytochemical or biological data, or non-original study design.

In total, 308 references were found, of which 49 were on PubMed, 124 were on Scopus, 71 were on Web of Science, and 64 were on SpringerLink. After removing duplicates (*n* = 57), article titles and abstracts were manually screened to exclude studies unrelated to the topic, as well as studies lacking data on the phytochemical profile of *O. spinosa*. A total of 70 articles were eliminated after this preliminary screening. The remaining 181 full-text documents were thoroughly analyzed. The following inclusion criteria were used in the selection process: studies published in English, full-text availability, and the presence of the term “*Ononis spinosa*” in the title and/or abstract. The exclusion criteria were as follows: the study topic (the absence of any information concerning the chemical composition, pharmacological activities or therapeutic properties of *Ononis spinosa*), conference papers, editorials, letters, short surveys, commentaries, case reports, abstracts published without a full article and records with no full text available. Studies focusing on other species or genera within the Fabaceae family were excluded. Although the primary search interval was 1997–2024, earlier studies with available full text were selectively included when they reported pharmacological or biological activities of *Ononis spinosa* that remain relevant and continue to be cited in the current literature, particularly in cases where more recent data are limited. Finally, 34 publications were considered for inclusion in our review. Figure 2 shows the study selection process.

Due to the narrative nature of this review, no formal quality assessment of the included studies was performed. However, the heterogeneity of study designs, extraction methods, and reporting standards was considered in the interpretation of the findings.

## 3. Phytochemistry and Biological Activities of *Ononis spinosa* Extracts

**Figure 3:**
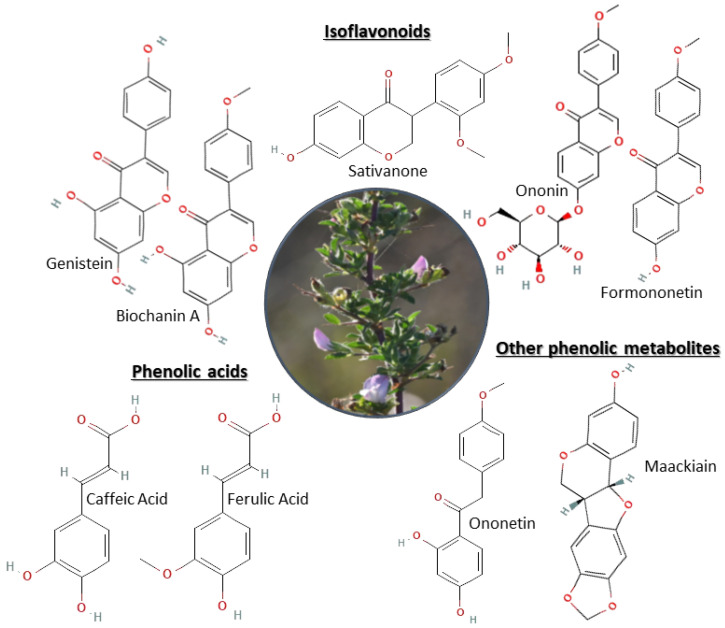
Phytochemical constituents identified in *Ononis spinosa* extracts. **Note:** Oxygen atoms are represented in red.

The phytochemical profile of *O. spinosa* is characterized by a complex and multifunctional spectrum of secondary metabolites, including phenolic compounds—such as flavonoids (particularly isoflavonoids, including isoflavones), phenolic acids, and triterpenes [17], which collectively contribute to a wide range of biological activities (Figure 3). Rather than being attributed to a single dominant compound, the pharmacological effects of *O. spinosa* may reflect the combined activity of multiple constituents [7,8,18,29]. This interpretation is supported by fractionation studies, in which the crude extract exhibited slightly higher activity than its corresponding fractions, suggesting a possible synergistic effect among the constituents; however, this interaction has not been directly demonstrated [18,29,30]. Consequently, its therapeutic effects can be interpreted as the outcome of the integrated activity of multiple constituents, consistent with the concept of a phytocomplex described for plant extracts [31]. This interpretation is consistent with studies on Fabaceae species, including *Ononis* taxa, which demonstrate that biological activity is closely related to the overall phytochemical composition and isoflavone content rather than a single isolated compound [8,30].

A detailed phytochemical analysis of a methanolic–aqueous extract of *O. spinosa* root using LC–MS/MS led to the identification of 34 isoflavonoid derivatives. These included representatives of several structural classes, such as isoflavones (e.g., formononetin and its glycosides), pterocarpans (e.g., maackiain and medicarpin derivatives), and dihydroisoflavones (e.g., sativanone and onogenin) [27].

Notably, several compounds, including calycosin derivatives and certain sativanone and onogenin derivatives, were reported for the first time in this species. Structural elucidation was based on characteristic fragmentation patterns obtained by MS/MS analysis and comparison with reference standards and literature data [27].

Another LC–MS/MS analysis of *O. spinosa* extracts revealed the presence of a series of nitrogen-containing isoflavonoid derivatives. Six compounds were identified, all corresponding to known isoflavonoid skeletons (including formononetin, pseudobaptigenin, sativanone, onogenin, medicarpin, and maackiain) conjugated with a nitrogen-containing moiety. These compounds were detected in low abundance, as indicated by their weak UV signals despite strong ionization in MS analysis. Their presence was confirmed across multiple samples, excluding the possibility of contamination [28].

While these findings expand the known phytochemical profile of *O. spinosa*, the study does not provide direct evidence of biological activity. Therefore, any pharmacological relevance of these compounds remains to be established in dedicated bioactivity assays.

**Figure 4:**
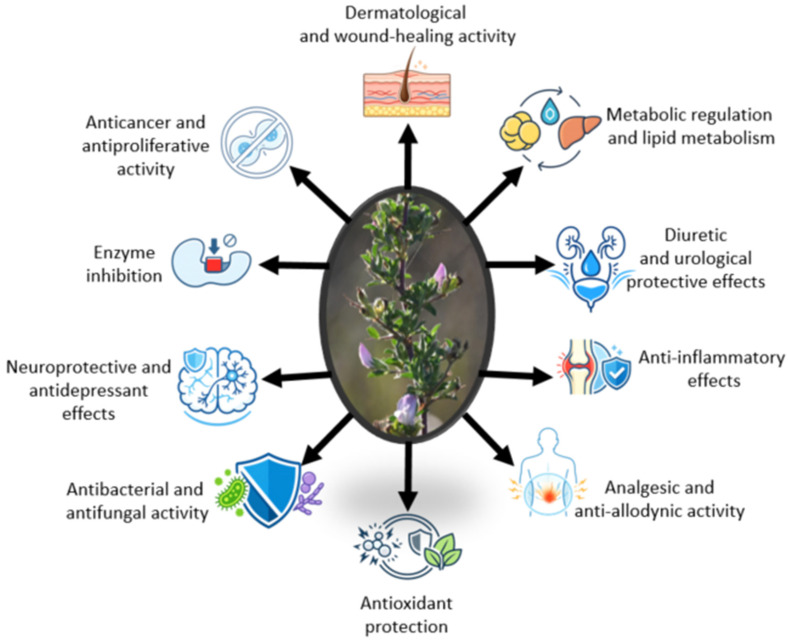
Schematic representation of the main biological activities of *Ononis spinosa* extracts across multiple organ systems. **Note:** Among the reported activities, anti-inflammatory, wound healing, analgesic, and antimicrobial effects are supported by more robust experimental evidence, particularly from in vivo studies using *Ononis spinosa* as a single agent. Detailed information is provided in Table 1.

For clarity and to provide an integrative overview, the principal biological effects across different organs and physiological systems of *O. spinosa* are summarized in Figure 4 and subsequently discussed in detail within the corresponding subsections.

Following the integrated overview presented in Figure 3 and Figure 4, the phytochemical composition and biological activities of *O. spinosa* extracts are discussed below in an activity-oriented manner. Each subsection focuses on a specific pharmacological effect, summarizing the main bioactive compounds involved, the experimental evidence available, and the proposed mechanisms of action.

### 3.1. Analgesic Activity

Transient receptor potential (TRP) channels are a large family of non-selective cation channels with high Ca^2+^ permeability, playing a central role in sensory processes such as pain and pruritus [32]. These channels act as molecular sensors capable of detecting a wide range of physical and chemical stimuli, including thermal, mechanical, osmotic, and chemical signals. Among the TRP subfamilies, the vanilloid subtype (TRPV), particularly TRPV1, has been extensively implicated in nociception and inflammatory pain mechanisms [33].

The analgesic potential of *O. spinosa* has been investigated in vivo using a capsaicin-induced mechanical allodynia model in rats. Intraplantar administration of a methanolic leaf extract significantly increased the paw withdrawal threshold at multiple time points, indicating a reduction in pain sensitivity. The attenuation of this effect by both a TRPV1 antagonist (BCTC) and a β_2_-adrenoreceptor antagonist (butoxamine) suggests the involvement of these pathways in the observed response. Complementary molecular docking analyses indicated potential interactions between selected constituents, such as campesterol, stigmasterol, and ononin, and the TRPV1 receptor; however, these in silico findings remain hypothetical and require experimental validation. Although the extract exhibited a stronger effect than diclofenac under the specific experimental conditions, direct comparisons with standard drugs should be interpreted cautiously due to differences in dosing and pharmacokinetic profiles [34].

In addition to TRPV1 modulation, the deoxybenzoin derivative ononetin, isolated from *O. spinosa*, has been shown to inhibit TRPM3 channels. Inhibition of TRPM3 has been associated with attenuation of thermal nociception in vivo in related flavanones [35].

Additional evidence of analgesic activity was obtained using an aqueous extract evaluated in a tail-flick assay. The extract produced significant analgesic effects at doses of 25, 50, and 100 mg/kg, with responses observed at multiple time points and, in some cases, comparable to those of reference drugs such as aspirin. However, comparisons with more potent opioids, such as morphine, remain limited by the simplicity of the experimental model [26].

Notably, in the same experimental setting, the extract did not demonstrate hepatoprotective effects in a CCl_4_-induced liver injury model. Instead, increased levels of liver enzymes (ALT, AST, and bilirubin), weight loss, and a case of mortality were reported, raising concerns regarding potential adverse effects under these conditions. This discrepancy between analgesic efficacy and lack of protective effects on liver function underscores the importance of a comprehensive evaluation of both pharmacological activity and safety [26].

Overall, available data support the in vivo analgesic potential of *O. spinosa* extracts in acute pain models, while highlighting the need for further studies to clarify the underlying mechanisms and to better define their safety profile.

### 3.2. Anti-Inflammatory Activity

Roots of *O. spinosa* have long been used in traditional medicine for their anti-inflammatory properties, an effect supported by several experimental studies [20]. Evidence from bioactivity-guided fractionation indicates that ethyl acetate extracts of *O. spinosa* subsp. *leiosperma* roots possess notable anti-inflammatory and wound healing activity in vivo. In particular, an active fraction (Fr-E5) increased tensile strength, reduced wound area, and elevated hydroxyproline levels, suggesting enhanced collagen deposition. In parallel, this fraction exhibited moderate anti-inflammatory effects in acute models, including acetic acid-induced capillary permeability and carrageenan-induced paw edema. However, no significant activity was observed in TPA-induced ear edema or in a chronic inflammation model (FCA-induced arthritis), highlighting a marked dependence on the experimental system. Isoflavonoid derivatives such as ononin and sativanone-7-O-glucoside were identified as potential contributors, showing moderate inhibition of hyaluronidase and elastase in vitro. Nevertheless, the slightly higher activity of the crude extract compared to its fractions suggests that the observed effects cannot be attributed to single compounds alone and may involve additive or synergistic interactions, which remain insufficiently characterized. Consistent with these findings, antioxidant activity was generally weak, indicating that the biological effects are unlikely to be driven primarily by redox mechanisms but may instead involve modulation of inflammation, extracellular matrix remodeling, and tissue repair processes [29].

Comparative studies across different *Ononis* species, plant parts, and extraction solvents further support these observations. Ethyl acetate root extracts consistently demonstrated the highest activity, emphasizing the importance of extraction conditions and plant material. At the same time, similar effects reported for related species, such as *O. natrix*, suggest that these properties may be shared at the genus level rather than being specific to *O. spinosa* [36]. However, variability between models remains a key limitation, with consistent activity observed mainly in acute inflammation, but not in chronic settings.

At the mechanistic level, additional in vitro evidence suggests that *O. spinosa* may interfere with inflammatory signaling pathways. A methanolic extract of the aerial parts inhibited cPLA2α activity in an enzymatic assay, indicating a potential effect on arachidonic acid metabolism, although the physiological relevance of this finding remains uncertain [24]. Moreover, a dichloromethane root extract demonstrated anti-inflammatory effects in LPS-stimulated human neutrophils, reducing TNF-α and IL-8 secretion and modulating adhesion molecules such as Mac-1 (CD11b/CD18) and CD62L. Mechanistic experiments suggested a possible involvement of TLR4-related pathways, including both direct cellular effects and indirect interactions with LPS. However, these findings remain indicative and do not establish a definitive mechanism of action [37].

Anti-inflammatory effects have also been investigated in models of lower urinary tract inflammation. Using isolated rat prostate tissue stimulated with lipopolysaccharide (LPS), an ex vivo model of prostatitis was employed to evaluate the effects of several plant extracts, including *O. spinosa*, *Solidago virgaurea*, *Phyllanthus niruri*, *Epilobium angustifolium*, and *Peumus boldus*, administered either individually or in combination as the dietary supplement Fluxonorm^®^ (OmegaPharma S.p.A., Cantù, Italy). Both the individual extracts and their combined formulation reduced markers of oxidative stress and inflammation in this model, including LPS-induced prostaglandin E_2_ (PGE_2_) and 8-iso-prostaglandin F_2_α (8-iso-PGF_2_α). However, phytochemical analysis indicated that *O. spinosa* extracts contained relatively low levels of common phenolic compounds such as gallic acid, catechin, and epicatechin, suggesting that these constituents are unlikely to be the primary drivers of the observed effects. Importantly, due to the multi-component design of the study, the specific contribution of *O. spinosa* to the overall anti-inflammatory activity cannot be clearly distinguished, and the results should therefore be interpreted with caution [38].

Clinical and preclinical data are also available for multi-component formulations containing *O. spinosa*. In a study investigating the dietary supplement Fluxonorm^®^, which includes several plant extracts, treatment resulted in reduced PC3 prostate cancer cell viability, decreased prostaglandin E_2_ (PGE_2_) release, and downregulation of COX-2 expression in vitro. In a clinical setting, administration of the formulation (1200 mg/day) was associated with improvements in lower urinary tract symptoms, including reduced International Prostate Symptom Score (IPSS), increased maximum urinary flow rate (Qmax), and improved quality of life (QoL). However, mechanistic interpretations in this study were largely based on compounds such as gallic acid, which are not characteristic constituents of *O. spinosa*. Therefore, the observed biological and clinical effects should be attributed to the combined phytocomplex rather than to *O. spinosa* alone, and the contribution of this species remains uncertain [39].

### 3.3. Antimicrobial Activity

Experimental investigations have demonstrated that *O. spinosa* extracts exert antimicrobial activity against a wide range of microorganisms, including both Gram-positive bacteria (*Streptococcus pneumoniae*, *Streptococcus pyogenes*, *Staphylococcus aureus*, *Staphylococcus epidermidis*, *Mycobacterium tuberculosis*, and *Mycobacterium avium*) and Gram-negative bacteria (*Klebsiella pneumoniae*, *Haemophilus influenzae*, *Pseudomonas aeruginosa*, and *Acinetobacter baumannii*). Nevertheless, the extracts generally display greater efficacy against Gram-positive microorganisms than against Gram-negative ones [40].

Among Gram-negative pathogens, *Escherichia coli* appears to be particularly susceptible to *O. spinosa* extracts. Inhibitory activity has been reported against several strains, including *E. coli* (ATCC 10536), *E. coli* (ATCC 25922), resistant *E. coli* isolates, and additional clinical variants such as PeruMycA strains [16,38,41]. As *E. coli* represents the most common causative agent of urinary tract infections, these findings support the traditional use of *O. spinosa* in urological disorders [42]. Furthermore, studies have shown that the extract can suppress colony development and reduce bacterial adhesion by preincubating uropathogenic *E. coli* or T24 human urinary bladder carcinoma cells with *O. spinosa* extract [41,43].

Another Gram-negative bacterium tested against *O. spinosa* extracts is *Pseudomonas aeruginosa* [40,41]. Minimum inhibitory concentration (MIC), minimum bactericidal concentration (MBC), and minimum bacteriostatic concentration (MBS) values of 64 µg/mL, 128 µg/mL, and ≥64 µg/mL, respectively, were reported by Orhan et al. [40]. However, conflicting results have also been described. In a study by Obistioiu et al. [41], three ethanolic extracts of *O. spinosa* (25%, 33%, and 40%) produced a stimulatory effect on the growth of *P. aeruginosa*, *Streptococcus pyogenes*, *Staphylococcus aureus*, and *Shigella flexneri* cultures grown on BHI agar, as reflected by negative values of bacterial inhibition rate (BIR) [41]. In the same study, inhibitory activity was nevertheless observed against *E. coli*, *Salmonella typhimurium*, and *Haemophilus influenzae*. The authors attributed these antimicrobial effects to the presence of polyphenolic compounds such as caffeic acid, quercetin, and kaempferol [41].

Regarding Gram-positive bacteria, *O. spinosa* extracts have demonstrated activity against several *Staphylococcus* species. Different strains of *Staphylococcus aureus*—including *S. aureus* (ATCC 6538), *S. aureus* (ATCC 25923), *S. aureus* (ATCC 11632), and methicillin-resistant *S. aureus* (MRSA isolates)—were shown to be sensitive to the extracts [16,38]. Another study reported a selective and relatively limited effect, with *O. spinosa* extract showing activity only against *Streptococcus pyogenes* (MIC 16 µg/mL), while no notable activity was observed against other tested microorganisms [40]. Nevertheless, contradictory findings were also reported by Obistioiu et al. [41], who observed a stimulatory effect on *S. aureus* ATCC 25923 colonies when a 40% extract was applied, reflected by strongly negative BIR values.

Early investigations into the antimicrobial activity of *O. spinosa* reported that crude extracts from the aerial parts exhibited moderate inhibitory effects against a range of Gram-positive and Gram-negative bacteria, as well as fungal strains. In particular, petroleum ether, ethanolic, aqueous, and butanol extracts demonstrated inhibition zones generally exceeding 8 mm in agar diffusion assays, suggesting a certain degree of antimicrobial potential. Among the tested extraction methods, butanol fractions showed comparatively higher activity.

However, the study employed relatively high extract concentrations (4 mg/disc) and relied on inhibition zone measurements rather than standardized minimum inhibitory concentration (MIC) values. In addition, the investigation included multiple plant species, and *O. spinosa* was not evaluated as an isolated case. Therefore, while these results indicate the presence of bioactive constituents with antimicrobial properties, they represent preliminary evidence and do not allow firm conclusions regarding potency or clinical relevance [44].

*O. spinosa* extracts have also shown inhibitory effects against other clinically relevant bacteria, including *Salmonella enteritidis*, *Salmonella typhimurium*, *Listeria monocytogenes*, *Listeria ivanovii*, *Micrococcus flavus*, *Klebsiella pneumoniae*, *Haemophilus influenzae*, *Acinetobacter baumannii*, and *Streptococcus* species such as *S. pneumoniae* and *S. pyogenes* [16,41].

Kırmızıgül et al. (1997) [45] isolated a novel glycoside, spinonin, together with the known isoflavonoid glycoside ononin and a pterocarpan derivative from *O. spinosa*. When evaluated in antimicrobial assays, spinonin and the related compounds exhibited only weak antibacterial activity against several bacterial strains, including *Pseudomonas aeruginosa* (e.g., MIC ~200 µg/mL). In addition, spinonin was tested for inhibition of HIV-1 reverse transcriptase but showed no detectable antiviral activity under the experimental conditions employed [45]. These findings highlight that the biological effects of individual isolated compounds may be modest and cannot be directly extrapolated to the pharmacological profile of the whole plant. Moreover, as these results are derived from isolated metabolites and a specific subspecies (*O. spinosa* subsp. *leiosperma*), they should be considered as indirect evidence and interpreted with caution when discussing species-level activity.

In addition to antibacterial effects, *O. spinosa* extracts also display antifungal activity. Inhibitory effects have been reported against several *Candida* species, including *Candida albicans*, *C. tropicalis*, *C. parapsilosis*, and *C. krusei*, as well as against dermatophytes such as *Trichophyton mentagrophytes*, *T. rubrum*, *Tr. tonsurans*, *Epidermophyton floccosum*, and *Microsporum gypseum* [38,40]. In one study, the strongest antifungal activity was observed against *C. krusei*, with MIC values of 32 µg/mL and MFC/MFS values of 64/32 µg/mL. Comparable activity was observed against *C. albicans* and *C. tropicalis*, while weaker activity was reported for *C. parapsilosis* [40].

Among dermatophytes, *Epidermophyton floccosum* appeared to be the most sensitive species to *O. spinosa* extracts, with an MIC of 64 µg/mL and fungistatic activity greater than or equal to 64 µg/mL. *Microsporum gypseum* and *Trichophyton rubrum* showed MIC values of 32 µg/mL and comparable fungistatic activity [40].

Although the present review primarily focuses on *O. spinosa*, limited data from other species within the genus *Ononis* may provide complementary insights into their biological potential. In this regard, *Ononis arvensis* has been investigated for its antimicrobial activity, revealing solvent-dependent effects against selected bacterial and fungal strains [46]. Specifically, chloroform and ethyl acetate extracts exhibited moderate inhibitory activity against microorganisms such as *Escherichia coli*, *Staphylococcus aureus*, and *Candida albicans*, whereas methanolic and aqueous extracts were largely inactive. However, the reported minimum inhibitory concentrations were considerably higher than those of standard antimicrobial agents, indicating relatively weak efficacy. These findings suggest that, while certain non-polar extracts of *Ononis* species may possess antimicrobial properties, their clinical relevance remains limited. Overall, such data support the notion that antimicrobial activity is not a predominant pharmacological feature of the genus, particularly when compared to its more consistently reported anti-inflammatory and analgesic effects [46].

Overall, the available evidence suggests that *O. spinosa* extracts may exhibit antimicrobial activity against selected Gram-positive and Gram-negative bacteria, as well as certain fungal strains. However, these findings are primarily derived from in vitro studies, often based on crude extracts or screening assays involving multiple plant species, and show considerable variability depending on the extraction method, plant material, and experimental conditions. In addition, some studies report limited, selective, or even absent activity, indicating that antimicrobial effects are not consistent across all models. While such observations point to the presence of bioactive constituents, the specific compounds responsible and their mechanisms of action remain insufficiently characterized. Therefore, although *O. spinosa* may represent a potential source of antimicrobial agents, the current evidence remains preliminary, and further well-designed studies are required to clarify its pharmacological relevance and reproducibility.

### 3.4. Antioxidant Activity

The antioxidant potential of *O. spinosa* extracts has been reported in several in vitro assays using different plant parts and extraction methods. Methanolic extracts obtained from the aerial parts exhibited measurable and dose-dependent antioxidant activity in multiple in vitro assays, including the phosphomolybdenum assay (1.50 mmol Trolox equivalents per gram of extract, mmol TE/g), DPPH (2,2-diphenyl-1-picrylhydrazyl radical scavenging assay; 23.96 mg TE/g), ABTS (2,2′-azino-bis(3-ethylbenzothiazoline-6-sulfonic acid) radical scavenging assay; 61.29 mg TE/g), CUPRAC (cupric ion reducing antioxidant capacity; 101.44 mg TE/g), FRAP (ferric reducing antioxidant power; 59.61 mg TE/g), and metal-chelating assays [16]. Further investigations on the methanolic extracts of *O. spinosa* subsp. *leiosperma* confirmed their antioxidant potential, demonstrating activity in DPPH and ABTS assays with IC_50_ values of 117.29 µg/mL and 127.25 µg/mL, respectively. Additionally, a 70% ethanolic extract inhibited the production of superoxide anion, with an IC_50_ value of 1.35 mg/mL [47].

Antioxidant activity has also been evaluated for individual compounds isolated from *O. spinosa*. Three constituents—puerol B (a phenolic compound), specionin (an iridoid glycoside), and a newly identified cyclic polyketide—were tested in antioxidant assays. Among them, puerol B showed the highest DPPH radical-scavenging activity (IC_50_ 0.09 ± 0.006 mg/mL), whereas specionin demonstrated the strongest ABTS scavenging capacity (IC_50_ 0.013 ± 0.0008 mg/mL). The newly identified δ-lactone displayed moderate antioxidant activity when compared with reference standards [48].

In contrast, an aqueous extract of *O. spinosa*, commonly used in traditional Turkish medicine, exhibited only limited antioxidant activity. A root decoction inhibited approximately 20.5% of DPPH radicals, a value considered insufficient to classify the extract as a strong antioxidant. This reduced activity is likely related to the relatively low total phenolic content of the extract (3.09 mg GAE/g) [40]. This finding suggests that antioxidant potential may not represent a dominant pharmacological property of *O. spinosa* extract under the tested conditions.

### 3.5. Cytotoxic Activity

Extracts of *O. spinosa*, particularly methanolic and ethyl acetate extracts obtained from aerial parts, have demonstrated cytotoxic effects against several cancer cell lines (e.g., MCF-7, MDA-MB-231), suggesting a potential role in cancer chemoprevention [16,31]. Methanolic extracts from the aerial parts inhibited the growth of breast adenocarcinoma MCF-7 cells (IC_50_ = 101.28 ± 8.29 µg/mL) and cervical carcinoma SiHa cells (IC_50_ = 181.96 ± 28.37 µg/mL), while also reducing the proliferation marker Ki67 in A172 glioblastoma cells at IC_50_ concentrations [16]. Cytotoxic activity has also been reported in related species of the genus *Ononis*. For example, ethyl acetate extracts obtained from the aerial parts (shoots) of *Ononis natrix* demonstrated inhibitory effects against MDA-MB-231 breast cancer cells (IC_50_ 29–41 µg/mL), suggesting a potential antiproliferative capacity at the genus level; however, such findings cannot be directly extrapolated to *O. spinosa* [49].

Additional evidence arises from studies employing a standardized multi-extract supplement (Fluxonorm^®^), which includes water-soluble *O. spinosa* extract. In human prostate cancer PC3 cells, the formulation reduced cell viability and suppressed PGE_2_ release and COX-2 expression [39]. These results suggest that *O. spinosa* may contribute to the antiproliferative activity of the phytocomplex, although its individual contribution cannot be separated from that of the other components.

### 3.6. Anti-Adipogenic Activity

The anti-adipogenic potential of *O. spinosa* has been explored in a single in vitro study using a hydroalcoholic root extract (50% aqueous methanol) and its major secondary metabolites, ononin (an isoflavone glycoside) and maackiain (a pterocarpan), in human Simpson–Golabi–Behmel syndrome (SGBS) adipocytes [50]. Adipogenesis is a process involving the coordinated activation of insulin-dependent phosphatidylinositol 3-kinase/protein kinase B (PI3K/AKT) signaling and downstream transcription factors such as CCAAT/enhancer-binding proteins (C/EBPs) and peroxisome proliferator-activated receptor gamma (PPARγ), which govern terminal adipocyte differentiation and lipogenesis [50,51,52].

Both ononin and maackiain significantly reduced lipid accumulation during adipocyte differentiation, whereas the crude root extract did not inhibit adipogenesis and, under the same experimental conditions, increased lipid accumulation. This contrast suggests that the observed biological effects may be primarily associated with specific secondary metabolites rather than the whole extract, highlighting the importance of compound-level investigations [50].

At the molecular level, the anti-adipogenic activity of these compounds appears to involve a multi-target mechanism acting across several stages of the adipogenic cascade. Early in the differentiation process, both ononin and maackiain were associated with the modulation of PI3K signaling, a key upstream regulator of adipogenesis initiation. This effect is supported by both in silico docking predictions and experimental evidence at the protein level. These findings suggest potential interference with insulin-mediated signaling pathways [50].

Downstream, both compounds modulated the expression of key transcription factors involved in adipocyte differentiation. Maackiain was associated with a marked reduction in adipogenesis by downregulating C/EBPα and PPARγ at both gene and protein levels, thereby suggesting suppression of terminal differentiation. In parallel, it reduced the expression of sterol regulatory element-binding protein 1 (SREBP1) and its downstream target acetyl-CoA carboxylase (ACC), suggesting inhibition of lipogenic pathways. These effects collectively suggest that maackiain may exert inhibitory effects on both adipogenesis and lipogenesis through disruption of the PPARγ/C/EBPα axis and associated metabolic pathways [50].

In contrast, ononin demonstrated a more moderate but consistent anti-adipogenic effect, associated with upregulation of sirtuin 1 (SIRT1) and modulation of PI3K signaling, together with suppression of PPARγ and adiponectin expression. Additionally, both compounds were associated with increased expression of AMP-activated protein kinase (AMPK), a central regulator of cellular energy homeostasis, suggesting a shift toward an anti-adipogenic metabolic profile [50].

Overall, these findings suggest that ononin and maackiain may act at multiple levels of the adipogenic cascade, including upstream signaling pathways (PI3K/AKT), transcriptional regulators (PPARγ, C/EBPα), and metabolic effectors (SREBP1, ACC), while also being associated with modulation of energy-sensing pathways (SIRT1, AMPK). However, the available evidence is currently limited to a single in vitro study supported in part by in silico analyses, without validation in in vivo or clinical models. Therefore, the anti-adipogenic potential of *O. spinosa* should be considered preliminary, and further studies are needed to clarify its potential translational relevance.

### 3.7. Antidepressant Activity

Ononin, an isoflavone glycoside identified in *O. spinosa*, has been associated with antidepressant-like effects supported by both in vitro and in vivo evidence [53]. Depression has been associated with impaired neuroplasticity and dysregulation of neurotrophic signaling, particularly involving brain-derived neurotrophic factor (BDNF), which plays a central role in neuronal survival, synaptic plasticity, and antidepressant responses [53,54].

In vitro studies using rat pheochromocytoma (PC12) cells suggested that a purified form of ononin promotes neurite outgrowth and neuronal differentiation in a dose-dependent manner, mimicking the effects of exogenous BDNF. These effects were accompanied by increased expression of neurofilament proteins and activation of key neurotrophic signaling pathways, associated with neuronal differentiation and survival, at both transcriptional and translational levels, as confirmed by RT-PCR and Western blot analyses [53].

At the molecular level, the antidepressant-like activity of ononin appears to be mediated through a coordinated activation of the BDNF–tropomyosin receptor kinase B (TrkB) signaling cascade. Ononin treatment significantly increased the expression of BDNF and its downstream receptor TrkB, leading to activation of the phosphatidylinositol 3-kinase/protein kinase B (PI3K/Akt) pathway and subsequent phosphorylation of cAMP response element-binding protein (CREB). This signaling cascade is known to regulate neurogenesis, synaptic plasticity, and neuronal survival, processes that are frequently disrupted in depression [55], suggesting that ononin may restore impaired neurotrophic signaling [53].

In addition to BDNF, ononin significantly upregulated other neurotrophic factors, including nerve growth factor (NGF) and glial cell line-derived neurotrophic factor (GDNF), which are involved in neuronal survival, differentiation, and synaptic maintenance, further supporting its role in promoting neuronal resilience and repair. These effects were consistently observed in both the frontal cortex and hippocampus, regions critically involved in mood regulation and frequently affected in depression [53].

The antidepressant-like effects of ononin were further supported in vivo using a chronic mild stress (CMS)-induced depression model in rats. Treatment with ononin (10–20 mg/kg) significantly improved depression-like behaviors, as evidenced by increased sucrose preference, reduced immobility time in the tail suspension test, and enhanced locomotor activity. These behavioral improvements were accompanied by restoration of BDNF, TrkB, PI3K, and CREB expression levels, as well as amelioration of stress-induced histopathological alterations in the hippocampus and frontal cortex [53].

Overall, these findings suggest that ononin exerts antidepressant-like effects through a multi-level mechanism involving activation of the BDNF–TrkB–PI3K/Akt–CREB signaling pathway and upregulation of neurotrophic factors, leading to enhanced neurogenesis and synaptic plasticity. However, the available evidence is derived from a single compound-based study and remains limited to preclinical models. To date, no evidence is available regarding the antidepressant activity of *O. spinosa* crude extracts or clinical validation; therefore, these findings should be interpreted with caution. Further studies, particularly in vivo and clinical investigations, are required to confirm these effects and assess their translational relevance.

### 3.8. Dermatological Activity

Dermatological applications of *O. spinosa* are supported by evidence from in vitro and in vivo studies, antimicrobial assays, ethnobotanical reports, and limited clinical evidence, consistent with both its traditional use and modern pharmacological potential in skin repair, inflammation control, and infection management.

Studies investigating the roots of *O. spinosa* have provided evidence for wound-healing ability [29,36]. In vivo wound closure was enhanced by the ethyl acetate extract obtained from the roots of *O. spinosa*, particularly its active fraction (Fr-E5), which contains isoflavonoids such as trifolirhizin, ononin, medicarpin-3-O-glucoside, onogenin-7-O-glucoside, and sativanone-7-O-glucoside. This fraction increased tensile strength by 33.4% and accelerated wound shrinkage by 51.4% by day 12. Collagen synthesis was increased, demonstrated by a hydroxyproline concentration of 30.9 ± 0.72 μg/mg [29]. In a comparative study investigating several *Ononis* taxa, the ethyl acetate extract obtained from the roots of *O. spinosa* subsp. *leiosperma* confirmed these effects in a comparable in vivo linear incision and circular excision wound models, showing a 42.6% increase in tensile strength and a 60.1% reduction in wound area by day 12, alongside significant fibroblast proliferation and collagen deposition on histopathological evaluation [36]. Collectively, available evidence suggests that *O. spinosa* root extracts may positively influence all major phases of wound healing, including inflammation modulation, proliferation, and remodeling.

Mechanistic investigations identified human hyaluronidase-1 (Hyal-1) inhibition as a potential mechanism through which *O. spinosa* extract may contribute to tissue regeneration. Hyal-1 degrades high-molecular-weight hyaluronic acid (HA), a key molecule for skin hydration, elasticity, and structural integrity. Strong Hyal-1 inhibitory activity was shown by dichloromethane extracts (IC_50_ 190 µg/mL), and the isolated compounds (sativanone, onogenin, medicarpin, and calycosin-D) indicate inhibition of 22–61%, with sativanone showing higher activity comparable to glycyrrhizinic acid [42]. Hyal-1 inhibition was also observed in a later study, where non-polar fractions achieved inhibitory values of 86% and 96% at 1 mg/mL; sativanone (isoflavone) remained the most active compound (IC_50_ ≈ 151 µM), reinforcing its role as a promising dermatological compound [56].

Enzyme-targeted effects were further investigated by in vitro assays showing that ononin and sativanone-7-O-glucoside exhibited inhibitory activity against both hyaluronidase and elastase, enzymes involved in the degradation of hyaluronic acid and elastin. Their observed inhibition (31–46%) suggests a potential contribution to the wound healing effects of *O. spinosa*, possibly through modulation of extracellular matrix remodeling processes [29].

The anti-inflammatory properties of *O. spinosa* are supported by in vitro studies showing that a dichloromethane root extract rich in isoflavonoids and triterpenes inhibits IL-8 and TNF-α release from LPS-stimulated human neutrophils. The extract also modulates immune-cell adhesion by decreasing CD11b/CD18 expression and increasing CD62L levels, changes associated with reduced leukocyte activation and migration. These effects may contribute to its anti-inflammatory activity, with evidence suggesting involvement of TLR4 signaling, potentially through both receptor antagonism and interactions with LPS [37].

Beyond wound healing and inflammation, several studies have reported antifungal and antibacterial activities of *O. spinosa* against fungal pathogens, including species associated with skin infections. Extracts inhibited dermatophytes such as *Trichophyton rubrum*, *Microsporum gypseum*, and *Epidermophyton floccosum* (MIC 16–64 µg/mL), and showed activity against yeasts including *Candida albicans*, *C. tropicalis*, and *C. parapsilosis* (MIC 16–32 µg/mL). Notably, the extract exhibited antifungal activity against *C. krusei* under the tested conditions [40]. Further studies reported broad-spectrum activity against Candida species, including antibiofilm effects, with activity in a similar range to fluconazole. Suggested mechanisms involve disruption of ergosterol biosynthesis and increased fungal cell membrane permeability. Importantly, the methanolic extract showed no cytotoxicity toward human cells, supporting its potential for further development as an antifungal agent [57].

Antimicrobial effects were also reported in earlier ethnopharmacological screenings, where *O. spinosa* extracts exhibited antibacterial activity against *Staphylococcus aureus* and *Bacillus cereus*, as well as antifungal activity against *Candida albicans*, Aspergillus flavus, and Fusarium moniliforme. The antifungal activity was described relative to the standard antifungal agent miconazole nitrate [44]. More recent studies reported antibacterial activity of *O. spinosa* extracts against resistant strains, including methicillin-resistant *Staphylococcus aureus* (MRSA), and showed marked inhibition of *Staphylococcus aureus* biofilm formation (up to ~80% at sub-MIC levels). Additionally, the extract significantly reduced staphyloxanthin production, a key virulence factor, indicating anti-virulence potential [16].

Ethnobotanical literature documents the use of *O. spinosa* in the management of skin conditions such as dermatitis and acne. In Eastern European ethnobotanical inventories, the species shows a Relative Dermatologic Importance (RDI) value of 19.02, reflecting its reported use in dermatological contexts [58]. Historical and ethnomedical sources also describe the use of *O. spinosa* in formulations intended for “blood purification,” a traditional concept generally associated with detoxification and elimination processes [59].

Some clinical data support its potential use in skincare. In a study involving 39 participants, a topical formulation containing *O. spinosa* root extract improved facial laxity and wrinkle depth. The study reported both immediate and long-term lifting effects (1.08 mm immediately; 1.80 mm after eight weeks). These results indicate its potential as an anti-aging ingredient and provide preliminary clinical support for its use among ethnomedicinal plants traditionally applied in dermatological conditions [56].

### 3.9. Diuretic Effects

The diuretic properties of *O. spinosa* extracts are supported by traditional usage records, biochemical studies, and limited pharmacodynamic evidence. Extracts from the roots of *O. spinosa* have long been used as weak diuretics to promote urine flushing and relieve minor urinary complaints, a practice consistently documented across several ethnobotanical regions. This traditional use is further supported by experimental findings demonstrating that root extracts inhibit human hyaluronidase-1 (Hyal-1), a mechanism associated with increased renal fluid excretion [42]. This traditional classification as a urinary-cleansing remedy is further supported by ethnobotanical data from Turkish folk medicine, where root decoctions of *O. spinosa* are administered twice daily for 5–10 days to induce diuresis and facilitate kidney stone elimination [60]. Comparable traditions appear in Polish ethnomedicine, where *O. spinosa* is recognized as a diuretic species [61], as well as in the Waldensian Alps, where root tinctures are used as depuratives, a category traditionally associated with purification and fluid elimination [62]. A similar ethnomedical role is reported in Iran, where root decoctions of *O. spinosa* are traditionally used for their diuretic effects and for the management of urinary tract inflammation [63].

Studies suggest that bioactive compounds (triterpenoid saponins, isoflavonoids and phenolic acids) from *O. spinosa* root extract may modulate renal water excretion, at least in part, through inhibition of human hyaluronidase-1 (Hyal-1), an enzyme involved in the degradation of high-molecular-weight hyaluronic acid (HA) in renal tissue [42]. Experimental evidence from renal physiology studies indicates that low-molecular-weight hyaluronic acid (LMW-HA) fragments, generated through Hyal-1-mediated degradation, can modulate renal fluid handling. In these studies, exogenous administration of LMW-HA or increased endogenous HA breakdown was associated with enhanced renal fluid excretion, possibly through alterations in tubular water permeability and interstitial matrix dynamics. Conversely, pharmacological inhibition of Hyal-1 has been linked to increased urine output, an effect attributed to the preservation of high-molecular-weight HA within the renal interstitium, which may influence water reabsorption processes. Bioactivity-guided fractionation of *O. spinosa* root extracts has indicated inhibitory activity against human hyaluronidase-1 (Hyal-1), and this mechanism has been proposed as a plausible explanation for the diuretic effects traditionally attributed to the plant [56].

Restharrow root preparations have shown moderate diuretic activity in rat models, providing limited but supportive pharmacological evidence for their traditional use [42]. This is further reinforced by regulatory recognition, as the European Medicines Agency acknowledges *O. spinosa* root preparations as herbal medicinal products intended “to increase the amount of urine to achieve flushing of the urinary tract,” particularly as adjuvants in minor urinary complaints [42].

*O. spinosa* is constantly referred to as a diuretic herb in other historical and regional sources. Urinary tract infections, renal inflammatory diseases, and dropsy have all been treated with infusions containing restharrow roots, leaves, or flowers [56]. The roots’ diuretic, “blood-purifying”, and expectorant properties are highly valued in Iraqi ethnomedicine [63], demonstrating the cross-cultural continuity of their use.

### 3.10. Urinary Tract Effects

Clinical, experimental, and ethnobotanical evidence across several independent studies support the potential involvement of *O. spinosa* in urinary system modulation. In a clinical evaluation of Fluxonorm^®^, a standardized multi-extract formulation containing *O. spinosa*, improvements were observed in men with lower urinary tract symptoms (LUTS), including increased maximum urinary flow and decreased IPSS and nocturia scores, while urinary pH and specific weight remained unchanged [39]. These effects have been attributed to the combined antioxidant and anti-inflammatory properties of the plant extracts; however, the specific contribution of *O. spinosa* remains difficult to isolate.

Further mechanistic and cellular evidence indicates that aqueous root extracts of *O. spinosa* exert pronounced anti-adhesive effects against uropathogenic *Escherichia coli*, significantly reducing bacterial adhesion to T24 bladder epithelial cells in a concentration-dependent manner and decreasing intracellular bacterial load by up to 69%, without affecting bacterial proliferation [43]. These findings suggest a potential protective role at the urothelial barrier level by limiting pathogen–host cell interaction, aligning with long-standing traditional use in urinary tract infections. Additional mechanisms involve modulation of inflammatory pathways, including inhibition of TLR-4-mediated IL-6 and TNF-α release, as well as suppression of Hyal-1 activity, both implicated in urothelial inflammation.

The use of *O. spinosa* in urinary problems is documented by several ethnopharmacological sources. Traditional use includes irrigation therapy for inflammatory diseases of the lower urinary tract and its supportive use in urinary tract infections [64], urinary tract inflammation, as reported in Iranian ethnomedicine [63], and the management of urinary tract infections and other inflammatory conditions of the urinary system in the Western Balkans, often in association with its diuretic use [65]. The species is also recognized in Polish ethnomedicine as a plant with urinary tract protective applications and urinary antiseptic properties [61]. Consistent with these reports, ethnobotanical data from Serbia indicate that *O. spinosa* roots prepared as an infusion are traditionally used to facilitate the elimination of kidney sand and small urinary stones [66].

### 3.11. Gastroprotective Effects

The gastroprotective properties of *O. spinosa* were evaluated in a rat model of ethanol-induced gastric ulcers. Methanolic leaf extract (0.5–1 g/kg) administered orally significantly decreased ulcer indices by 80.39% and 98.71%, respectively, outperforming esomeprazole. Histological analysis showed reduced leukocyte infiltration, hemorrhage, submucosal edema, and mucosal necrosis in extract-treated groups. Treatment also increased COX-2 expression and gastric glutathione levels, suggesting the involvement of prostaglandin-mediated cytoprotective mechanisms and antioxidant activity. LC–MS analysis identified ononin as the major compound, followed by trifolirhizin, myricitrin, gentisic acid, cycloartenol, and quercetin [67].

### 3.12. Hepatoprotective Activity

The hepatoprotective potential of *O. spinosa* remains limited. In a CCl_4_-induced liver injury model, administration of an aqueous extract (100 mg/kg) did not result in significant reductions in serum liver enzyme levels (aspartate aminotransferase (AST) and alanine aminotransferase (ALT)) nor improve histopathological alterations compared to the untreated CCl_4_ group [26]. These findings indicate the absence of a hepatoprotective effect and suggest a possible worsening of biochemical parameters under the tested conditions.

The interpretation of the reported biological activities may be influenced by considerable heterogeneity across studies, including differences in extraction methods, plant material used, and experimental models. In particular, the lack of standardization regarding solvent systems, extract concentrations, and the reporting of IC_50_ and MIC values may limit the comparability of results. Additionally, some discrepancies in reported activities could be attributed to methodological variability rather than true biological differences. These aspects highlight the need for more standardized and rigorously designed studies in future research. Therefore, such limitations should be taken into account when interpreting the therapeutic potential of *O. spinosa*.

To facilitate an integrated synthesis of the extensive experimental and mechanistic evidence discussed above, the principal bioactive compounds of *O. spinosa* and the main phytochemical fractions reported in the literature were summarized in Table 1. The table provides a consolidated overview of the most relevant constituents, the plant parts and extraction approaches employed, and their associated biological activities across different organ systems, together with the level of experimental or clinical evidence. This summary is intended to support comparison across studies and to highlight the phytochemical basis underlying the diverse pharmacological effects described in this section.

**Table 1:** Comprehensive overview of the pharmacological activities of *Ononis spinosa* extracts and isolated compounds, including experimental models, extract types, and levels of evidence.

Reported Biological Activities	Plant Part	Type of Extract	Compound	Experimental Model	Dose/Concentration	Evidence Level	Reference
Analgesic	Not mentioned	Aqueous extract	-	Rat model, tail-flick assay	Doses of 25, 50, 100 mg/kg,	In vivo	[26]
Analgesic	Leaves	Methanolic extract	-	Capsaicin-induced mechanical allodynia model in rats		In vivo	[34]
Anti-inflammatory	Aerial parts	Methanolic extract	-	Inhibitory activity against cPLA2α, IC_50_ = 39.4 ± 6.49 µg/mL		In vitro	[24]
Anti-inflammatory, wound healing	Roots	Ethyl acetate fraction (Fr-E5)	Isolated ononin and sativanone-7-O-glucoside	Wound models, acetic acid-induced capillary permeability and carrageenan-induced paw edema models		In vivo	[29]
	Roots	Ethyl acetate extract	-	Wound models, acetic acid-induced capillary permeability and carrageenan-induced paw edema models		In vivo	[36]
Anti-inflammatory	-	Fluxonorm^®^	-	Isolated rat prostate tissue		Ex vivo	[38]
		Fluxonorm^®^	-	Clinical study involving 30 patients with lower urinary tract symptoms		Clinical	[39]
Antimicrobial	Aerial parts	Petroleum ether extract Ethanolic extract Aqueous extract Butanol fraction	-	Agar diffusion assay		In vitro	[44]
	Not mentioned	Aqueous ethanolic extract	-	Disc diffusion assay		In vitro	[22]
	Aerial parts		Isolated spinonin	Minimum inhibitory concentration (MIC) assay		In vitro	[45]
	Not mentioned	Aqueous extract	-	MIC determination microdilution	Stock solution: 512 µg/mL. Serial dilutions: 256–0.06 µg/mL	In vitro	[40]
	Aerial parts	60% Ethanolic extract		MIC determination (microdilution)	25, 50 and 100 µg/mL	In vitro	[41]
Antioxidant	Not mentioned	Aqueous extract	-	DPPH radical scavenging assay	-	In vitro	[40]
	Aerial parts	Methanolic extract	-	DPPH assay FRAP assay CUPRAC assay ABTS assay	-	In vitro	[16]
	Aerial parts	75% Ethanolic extract		In vitro: superoxide radical scavenging assay using the xanthine/xanthine oxidase system Ex vivo: lipid peroxidation assay in rat liver homogenate induced by FeCl_2_–ascorbic acid	Superoxide radical scavenging assay: 0.5–10 mg/mL Lipid peroxidation assay: 2.5–10 mg/mL	In vitro Ex vivo	[47]
	Whole plant	Extraction with ethanol, subsequent fractionation (chloroform, n-hexane, methanol-water)	Isolated puerol B, specionin	DPPH assay ABTS assay	Puerol B: IC_50_ = 0.09 mg/mL (DPPH) specionin: IC_50_ = 0.013 mg/mL (ABTS)	In vitro	[48]
Cytotoxic	Aerial parts	Methanolic extract		Crystal violet assay—MCF-7 breast cancer line	50–250 µg/mL	In vitro	[16]
Anti-hyaluronidase	Roots	Extracts obtained using solvents of different polarity: -dichloromethane (most active); -acetone–water (7:3, *v*/*v*); -ethanol; -ethanol–water (1:1, *v*/*v*); -methanol; -hot water.	Isolated onogenin, sativanone, medicarpin, calycosin-D	Enzymatic assay using hyaluronidase (Hyal-1)	Sativanone: 250 μM → 61.2% inhibition IC_50_ = 150.7 μM	In vitro	[42]
Anti-adipogenic	Roots	50% aqueous methanolic extract	Isolated ononin and maackiain	Human Simpson–Golabi–Behmel syndrome (SGBS) adipocytes	Extract: 5, 10, 25 µg/mL Ononin: 5, 10, 25 μM Maackiain: 5, 10, 25, 50 μM	In vitro In silico predictions	[50]

The added value of the present review lies in the integrative synthesis of phytochemical and pharmacological data, which have previously been reported in a fragmented manner across the literature. Unlike previous reviews, this work systematically correlates specific phytochemical classes (e.g., isoflavonoids, phenolic acids) with their reported biological activities, allowing for a more functional interpretation of the available evidence. Isoflavonoids, flavonoids, and other phenolic compounds identified in *O. spinosa* belong to a broader class of secondary metabolites characteristic of the Fabaceae family [8,68,69,70]. This observation is consistent with the well-documented phytochemical diversity of legumes, in which flavonoids are widely distributed, while some classes of secondary metabolites tend to show a more restricted and taxon-dependent occurrence [68]. From a biosynthetic perspective, the occurrence of these compounds is linked to enzymatic pathways involved in isoflavonoid production, which are well documented in leguminous plants [70]. Consequently, these findings support the existence of a shared metabolic framework at the family level, although species-specific variations may still occur. Although comparative phytochemical data across *Ononis* species remain limited, the available evidence suggests that related taxa may share similar classes of secondary metabolites, warranting further investigation.

## 4. Conclusions

The available evidence indicates that *O. spinosa* is a phytochemically complex species characterized mainly by the presence of polyphenols (flavonoids/isoflavonoids/pterocarpans, phenolic acids) and triterpenes, which collectively appear to contribute to a broad spectrum of biological activities. Experimental data support the anti-inflammatory, antimicrobial, antioxidant, wound-healing, diuretic, and urological effects of the extracts, with additional evidence suggesting antiproliferative, neuroactive, and metabolic regulatory properties. Many of these effects appear to be mediated through enzyme inhibition (e.g., hyaluronidase, cPLA2α, COX pathways), modulation of inflammatory signaling, and interference with microbial adhesion and oxidative stress. However, most findings derive from in vitro and preclinical models, while clinical evidence remains limited. Future research should aim to clarify the contribution of individual compounds versus whole extracts, standardize extraction protocols, and establish clinically relevant dosing and safety profiles. Among the reported activities, particular attention should be given in future studies to the anti-inflammatory, urological, and wound-healing effects, which are supported by relatively more consistent experimental evidence and align with the traditional uses of the plant. These areas may represent the most promising directions for translational and clinical research. A more robust translational framework is necessary to better define the therapeutic value of O. spinosa in modern phytotherapy, as it is a species with a complex chemical composition and a therapeutic potential that warrants further investigation.

## Data Availability

No new data were created or analyzed in this study. Data sharing is not applicable to this article.
